# Decoding Immune Heterogeneity of Triple Negative Breast Cancer and Its Association with Systemic Inflammation

**DOI:** 10.3390/cancers11070911

**Published:** 2019-06-28

**Authors:** Sandra Romero-Cordoba, Elisabetta Meneghini, Milena Sant, Marilena Valeria Iorio, Lucia Sfondrini, Biagio Paolini, Roberto Agresti, Elda Tagliabue, Francesca Bianchi

**Affiliations:** 1 Biochemistry Department, Instituto Nacional de Ciencias Médicas y Nutrición Salvador Zubirán, Mexico City 14080, Mexico; 2 Molecular Targeting Unit, Department of Research, Fondazione IRCCS Istituto Nazionale dei Tumori, 20133 Milan, Italy; 3 Analytical Epidemiology and Health Impact Unit, Department of Research, Fondazione IRCCS Istituto Nazionale dei Tumori, 20133 Milan, Italy; 4 Dipartimento di Scienze Biomediche per la Salute, Università degli Studi di Milano, 20133 Milan, Italy; 5 Pathological Anatomy Unit, Department of Pathology and Laboratory Medicine, Fondazione IRCCS Istituto Nazionale dei Tumori, 20133 Milan, Italy; 6 Division of Surgical Oncology, Breast Unit, Fondazione IRCCS Istituto Nazionale dei Tumori, 20133 Milan, Italy

**Keywords:** TNBC, immune heterogeneity, TILs, inflammation, PLR, platelet, CBC

## Abstract

Triple negative breast cancer (TNBC) is an aggressive subtype with limited therapeutic options. New opportunities are emerging from current comprehensive characterization of tumor immune infiltration and fitness. Therefore, effectiveness of current chemotherapies and novel immunotherapies are partially dictated by host inflammatory and immune profiles. However, further progress in breast cancer immuno-oncology is required to reach a detailed awareness of the immune infiltrate landscape and to determine additional reliable and easily detectable biomarkers. In this study, by analyzing gene expression profiles of 54 TNBC cases we identified three TNBC clusters displaying unique immune features. Deep molecular characterization of immune cells cytolytic-activity and tumor-inflammation status reveled variability in the local composition of the immune infiltrate in the TNBC clusters, reconciled by tumor-infiltrating lymphocytes counts. Platelet-to-lymphocyte ratio (PLR), a blood systemic parameter of inflammation evaluated using pre-surgical blood test data, resulted negatively correlated with local tumoral cytolytic activity and T cell–inflamed microenvironment, whereas tumor aggressiveness score signature positively correlated with PLR values. These data highlighted that systemic inflammation parameters may represent reliable and informative markers of the local immune tumor microenvironment in TNBC patients and could be exploited to decipher tumor infiltrate properties and consequently to select the most appropriate therapies.

## 1. Introduction

Triple negative breast cancer (TNBC) represents a minority (10–17%) of all breast carcinomas (BC) [1], however, it stands for an important challenge in actual clinical practice due to the poor clinical outcomes compared with non-TNBC [2,3]. It is known that tumor progression and subsequent metastasis may be impaired by an effective antitumor immune response. Notably, growing evidence shows a highly diverse immunogenic activity across breast cancer subtypes that may be correlated with phenotypic heterogeneity of breast cancer [4,5,6,7]. Several studies have explored the biological and clinical significance of immune system in TNBC [8,9,10,11,12]. TNBC is associated with high density of tumor infiltrating lymphocytes (TILs) defined by histopathology evaluation, which represents a robust intratumoral inflammatory response describing triple negative (TN) tumors as an immunogenic neoplasia [4,5,6]. Diverse evidence indicated that tumor-associated inflammatory cells improve survival in BC, including TNBC [4,7,13,14]. An enhanced innate and adaptive immunity, as revealed by TILs count, improves chemotherapy [14,15] and radiotherapy responses, regardless of breast cancer subtype [16].

Nowadays, besides count of TILs and immunoscoring of T-cell subpopulations by immune pathological evaluation, other methods recently emerged to assess tumor immune landscape such as deconvolution approaches that allow to define the proportion of immune cells infiltrating the tumors [17], and gene-expression signatures that mirror the immune-state of the neoplasia and its microenvironment [18,19,20,21]. Molecular phenotyping identified tumor immune response genes as key component in the pathobiology of TNBC [22,23,24,25]. These findings have encouraged the use of immunomodulatory agents in ongoing TNBC clinical trials [9,26,27] and pointed the emergency of a deep characterization of TNBC immune-related features and their dynamics across tumor life-time, to provide new immune markers.

A chronic systemic inflammatory response is defined as an aberrantly prolonged form of protective responses against tissue homeostasis disruption [28]. Elevated systemic inflammation is consistently associated with poor outcome in many solid tumor [29,30], including TNBC breast cancer [31]. Particularly, count of white cells derived from peripheral blood cell test (complete blood count, CBC), including neutrophils, lymphocytes, platelet cells and their proportions such as neutrophil to lymphocyte ratio (NLR) and platelet to lymphocyte ratio (PLR), were found effective predictors of BC poor survival outcome [31,32,33]. For instance, PLR represented a prognostic marker for overall survival (OS) in patients who receive chemotherapy [34]. In addition, in neoadjuvant therapeutic scheme, complete pathological response was significantly higher in breast cancer patients with low PLR [35], and in hormone-receptor-negative (HR-) breast cancer increased PLR associated with poor survival [36].

Nevertheless, although there is evidence of the prognostic power of systemic hematological cell count, the relationship between local and systemic inflammatory responses in BC is still unknown. In other solid tumor types, parameters of local and systemic inflammation appeared to be independent from each other (i.e., in colon cancer) or inversely associated (i.e., in laryngeal squamous cell carcinoma) [37,38]. Although recent studies demonstrated that tumor eradication via immunotherapy requires peripheral immune cell activity [39], the association between systemic inflammation and local immune response in BC remains unexplored. Clearly, there is a major need to better understand the genomic, molecular, and biological immune landscapes of TNBC and to dissect the relationship with systemic inflammation to finally propose novel markers to track immune states and their evolution along cancer-time by accessible circulating molecules.

Here, using unsupervised clustering analysis based on immune-related gene expression signatures derived from the literature, we demonstrated the existence of three immuno-clusters in TNBC tumors. We also showed an association of tumor immune infiltrate features with the systemic hematological PLR parameter. This evidence provides a proof-of-concept of the informative value of PLR as a surrogate metric of local tumor immune landscape of TNBC patients, which may represent an accessible tool, useful for real time monitoring of immune activation at tumor site.

## 2. Results

### 2.1. Comprehensive Genomic Characterization of Immune-Cell Infiltration in Triple Negative Tumors Identifies Three Immuno-Clusters that Portray Different Immune-Landscapes

**Figure 1:**
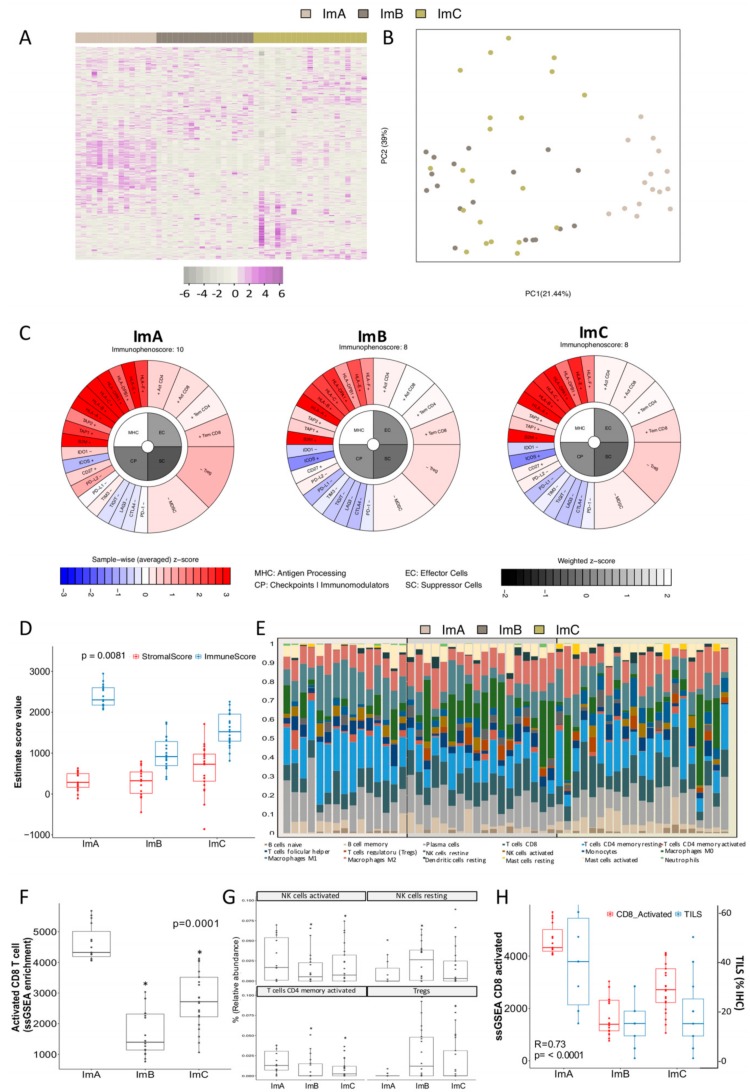
Immune landscape of the three immuno-clusters identified in triple negative tumors. (**A**) Heatmap of the expression profiles of the immune-related genes (*n* = 708) evaluated across the immuno-clusters (Im-Clus) defined (ImA, Imb and ImC). Violet indicates elevated gene expression while gray indicates reduced gene expression. (**B**) Principal component analysis (PCA) of the Im-Clus computer as a linear combination of the immune-related gene. Component 1 and 2 explain 60% of the variance. (**C**) Visual representation of computed immunoscores for each Im-Clust in an immunophentogram of the four immunogenicity categories of the tumors. (**D**) Boxplot of immune and stromal score distribution assessed by Estimated algorithm. (**E**) Relative abundance fractions (%) of immune cell population in individual tumors belonging to each of the Im-Clus using CIBERSORT tool. (**F**) Activated CD8+ T cells ssGSEA score values among the im-Clus. (**G**) Relative median infiltration of NK activated and resting cells, as well as CD4+ T cells and Tregs across the most significant altered immune-infiltrated population in the Im-Clus (ssGSEA of immune-related gene terms). (**H**) Technical validation and comparison between individual enrichment scores computed by ssGSEA on CD8+ (red boxplot) activated gene set and tumor infiltrating lymphocytes (TILs) pathological evaluation by hematoxylin/eosin evaluation (blue boxplot). * *p*-value ≤ 0.05.

By applying the non-negative matrix factorization (NMF) algorithm on gene expression profile of annotated immune related-genes [17,19] on 57 TNBC, tumors 5 immuno-clusters (Im-Clus) were defined (Figure S1A). To get more reliable mathematical and biological groups, 54 TNBC tumors were re-classified into 3 Im-Clus (ImA *n* = 15, ImB *n* = 18, ImC *n* = 21) (Figure 1A) (See section of Materials and Methods for details, Figure S1B,C). Principal component analysis (PCA) demonstrated robust differences in the expression portraits between the 3 Im-Clus identified by the NMF clustering (Figure 1B). The standard clinical and pathological characteristics of our cases were described in Table S1. The majority of women were ≥50 years of age (63%) and young patients were less likely to belong to ImC (ImA 53.3%, ImB: 44.4%; ImC: 19.1%; *p* = 0.084).

To decipher the immune heterogeneity among the established Im-Clus, we took advantage of immune-related tools recently published in the literature. First, to investigate whether the Im-Clus show different immunophenotypes, an in silico immunophenoscore [19] was computed. Immunophenogram of ImA presents an enrichment in tumor immune-intrinsic factors, such as immunoinhibitors (e.g., programmed death-ligand 1, PD-L1) and major histocompatibility complex class molecules (e.g., HLA, TAP1), as well as an enhancement in effector cells (e.g., activated CD8 T cells (CD8+) and activated memory CD4 T cells (CD4+)), which results in a immunophenoscore of 10. Instead, ImB and ImC presented a lower enrichment of antigen processing machinery, check points immunomodulators and effector cells, which together define an immunophenoscore of 8 (Figure 1C), meaning that ImA seems to be the most immunogenic group, in comparison to ImB and C. 

We then examined the distribution of stromal and immune content per Im-Clus by computing Estimate algorithm [21]. ImA was characterized by a significantly higher immune cells scores than the rest of the Im-Clus (*p* = 0.008), whereas ImB and mainly ImC showed a similarly distribution of stromal and immune scores (Figure 1D). Collectively, these results confirmed that ImA tumors tend to have stronger tumor immunity among the Im-Clus evaluated. In addition, when comparing tumor purity, we observed a descending pattern from ImC to ImA cluster (*Kruskal–Wallis* test, *p* ≤ 0.05) (Figure S2A). The median tumor purity of all evaluated cases computed with Estimated was 61%, in accordance to our sample inclusion criteria (Figure S2A). These results suggest that ImA malignancies contain the highest number of immune cells and ImC contain the highest number of tumor cells and stromal cell components. 

Composition of infiltrating immune cells was examined by CIBERSORT method [17] and ssGSEA of immune-related gene terms [40]. Each Im-Clus presented a unique content and mixture of immune infiltrating cell populations (Figure 1E). ImA, the more immune-active group, showed the higher enrichment of CD8+ (Figure 1F), as well as a higher relative abundance of other immune-active cells such as natural killer cells (NK) and effector CD4+ [41] (Figure 1G); while the immune-suppressive subtypes, ImB and ImC, were enriched in regulatory T cells (Tregs) (Figure 1G). Moreover, the percentage of samples presenting Tregs, NK and memory B cells was lower in tumors classified as ImA than ImB and ImC (Figure S2B), whereas neutrophils were present in a higher percentage of tumors belonging to ImA (Figure S2B).

To assess whether immune phenotypes based on gene expression reflect pathological evaluations of tumor infiltrating immune cells, tumor infiltrating lymphocytes (TILs) were evaluated. There was a strong significant correlation between the percentage of TILs and the enrichment score of activated CD8+ T cells (Spearman’s ρ = 0.728, *p* < 0.0001) (Figure 1H). A proportion of TILs equal to or higher than 20%, a threshold reported to have a clinical relevance [42,43], occurred in 93% of ImA tumors versus 33% and 47% of ImB and ImC tumors, respectively (Figure S2C,D).

**Figure 2:**
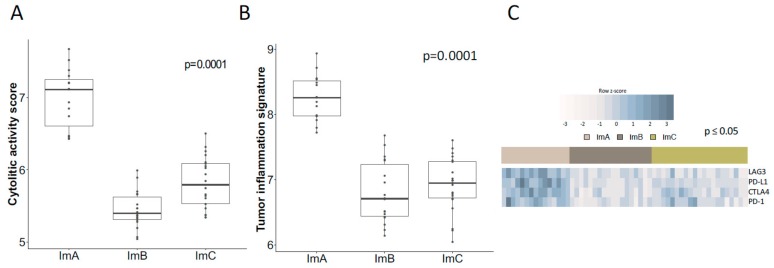

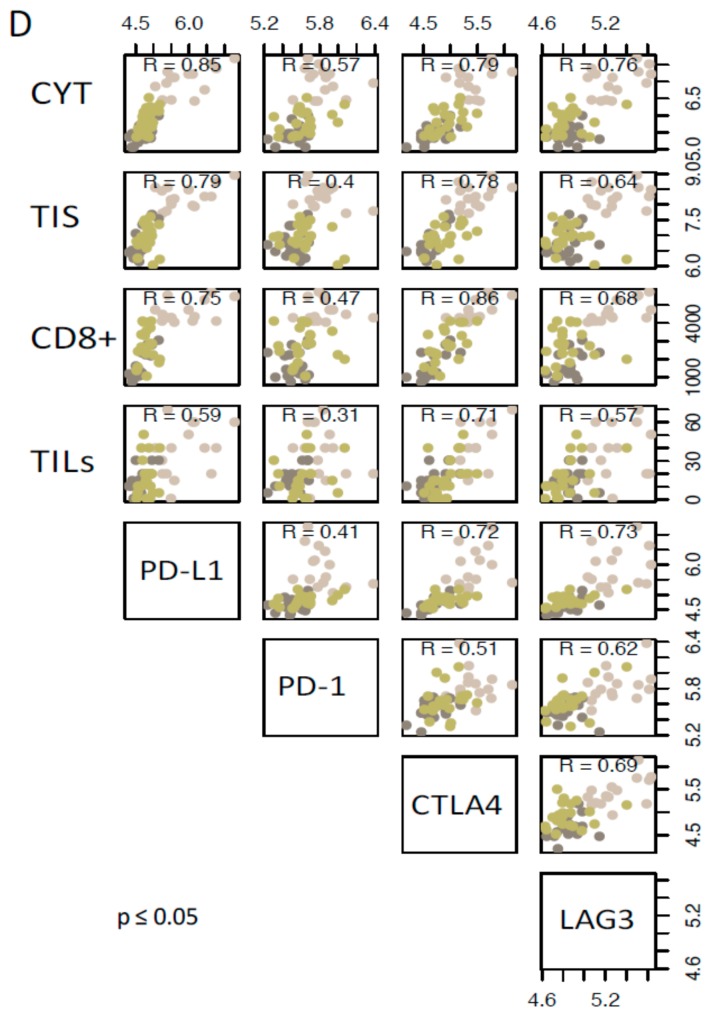
Local immune microenvironment and immuno-inhibitors portrait among the immune-clusters. Comparison of (**A**) Cytolitic activity score and (**B**) Tumor inflammation signature score values presented in boxplots. (**C**) Heatmap of gene expression of immune inhibitory molecules among the three immune clusters. Blue represent up-regulation while gray down-modulation. (**D**) Correlation matrix of local immune features and immune-inhibitory molecules across the Im-Clus, as measured by Spearman R coefficients. CD8+: ssGSEA enrichment score of activated CD8 t cells term; TILs: Pathological evaluation of the percentage of tumor infiltrating lymphocytes. All presented correlations are significant with *p* values ≤ 0.05. Statistical comparison based on Kruskal-Wallis method.

Since effective natural anti-tumor immunity requires a cytolytic immune response, cytolytic activity (CYT) was analyzed in our TN cases. ImA exhibited significantly higher CYT score compared to ImB and ImC (median: 7.11 vs. 5.40 and 5.79, *p* = 0.0001) (Figure 2A). Further, to explore a pre-existing adaptive immune response within tumors, we computed the Tumor Inflammation Signature (TIS) algorithm [18]. ImA presented the highest TIS score (median: 8.26 vs. 6.71 and 6.95, *p* = 0.0001) when compared to ImB and ImC (Figure 2B), consistent with the elevated CYT described in this immune-cluster.

To identify the dominant determinants of immune cell infiltration in the Im-Clus, the expression of the immune inhibitors Programmed Cell Death 1, Programmed Cell Death 1 Ligand, Cytotoxic T-Lymphocyte Antigen 4 and Lymphocyte Activation protein 3 (PD-1/PD-L1/CTLA4/LAG3) was analyzed. This molecular axis resulted up-modulated in the ImA cluster in comparison to ImB and C (Figure 2C). Likely, a strong correlation between most of these immune inhibitory genes, CYT/TIS scores and the proportion of CD8+ T cells (ssGSEA CD8+ and TILs) was observed (Figure 2D), suggesting potential coordinated mechanisms and biological signaling pathways that maintain the immune surveillance or the immune evasion in each Im-Clus. Collectively, these analyses identified an extensive immune heterogeneity in TNBC and revealed three immune-clusters that represent unique biological entities with specific immune-molecular features, as illustrated by the assorted cytolytic activity and inflammatory phenotypes identified, as well as the distinct immune cell populations infiltrating the tumoral cells.

### 2.2. Inflammation and Tumor Immune-Features Are Correlated within TNBC Immune-Clusters

**Figure 3:**
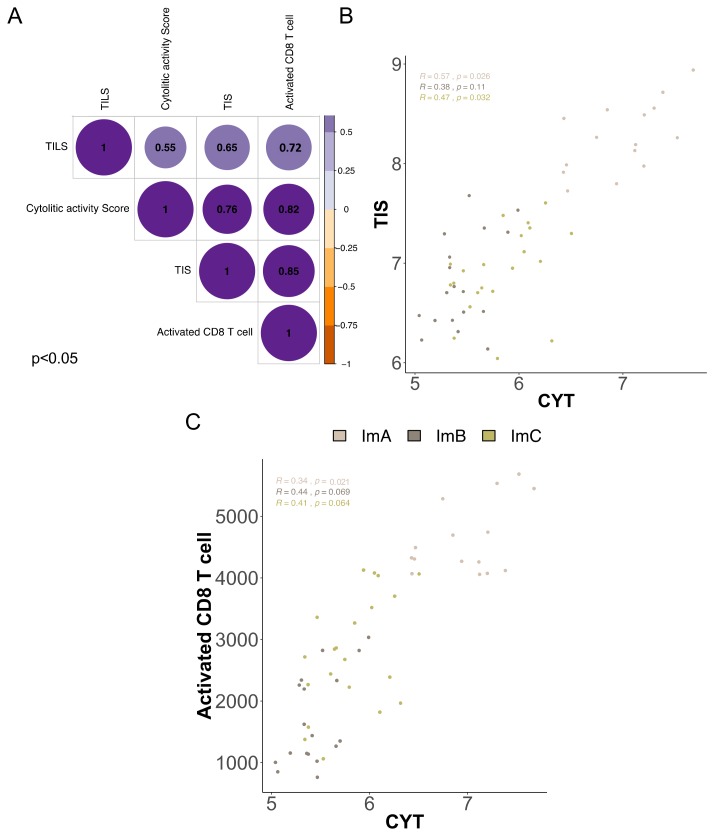
Correlation between local immune features among Im-Clus. (**A**) Matrix correlation of triple negative tumors among local immune features. The bubble color corresponds to the correlation direction. The number presented corresponds to the R value. All correlations presented are significant with *p* value < 0.05. Scatter plot of (**B**) cytolytic activity (CYT) vs. Tumor Inflammation Signature (TIS) and (**C**) CYT vs. activated CD8+ T cells and their correlation value and statistical significance reported as *p* value. Each Im-Clus is reported by different colors as indicated in the legend. All R coefficients are measured by Spearman method.

To identify cooperative immune phenotypes and functions, we determined the correlation between immune tumoral features in overall TNBC cases. We found a significant positive correlation between the CYT score, CD8+ infiltration and inflammation status (TIS) (Figure 3A), suggesting that CD8+ infiltration results in the activation of immune effectors, consistent with previous reports. [44,45]. Correlation analyses within each Im-Clus revealed heterogeneous association patterns. CYT positively correlates with TIS in ImA and C sub-groups and no correlation was observed in ImC (Figure 3B). CYT and activated CD8+ T cell were correlated only in ImA tumors (Figure 3C). These data are consistent with our previous data underlying varied features of local immune microenvironment among TNBC, and indicate the correlation of immune regulatory responses and mechanisms and the relevance of specific immune features of each immune-cluster.

### 2.3. The Systemic Inflammatory Marker Platelet-to-Lymphocyte Ratio Correlates with Local Immune Status of the Immune-Clusters 

**Figure 4:**
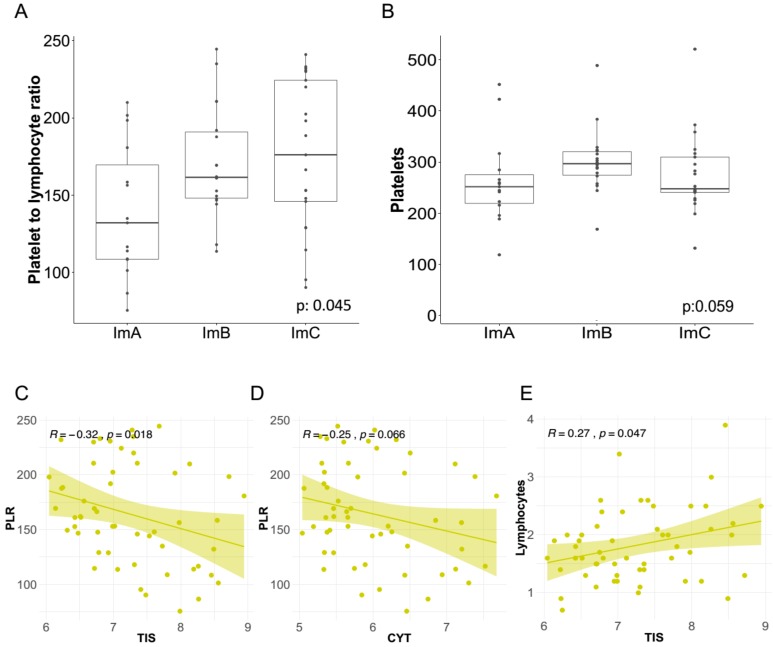
Correlation of local immune features and the inflammatory systemic blood markers in the triple negative breast cancer cases. Distribution of (**A**) platelet-to-lymphocyte ratio (PLR) and (**B**) platelets acquired through blood sample test in the Im-Clus. Scatter plots showing the correlation of: (**C**) Tumor Inflammation Signature (TIS) vs. PLR, (**D**) CYT vs. PLR, and (**E**) TIS vs. lymphocytes. All R coefficients measured by Spearman method. Statistical comparison based on Kruskal-Wallis method.

We investigated the relationship between the systemic levels of inflammatory parameters and the TNBC Im-Clus. Notably, the median value of the systemic inflammatory marker PRL was significantly lower in ImA than in the other Im-Clus (132.2 vs. 161.6, 176.2; *p* = 0.045) (Figure 4A), similarly median platelets count was lower in the ImA than in the other Im-Clus (*p* = 0.059) (Figure 4B). Likewise, in multinomial logistic regression analysis taking ImA as a reference group, PLR counts were still significantly associated with the Im-Clus (age-adjusted OR 1.20, 95% confidence interval (CI) 1.00–1.44 for ImB and 1.24, 95% CI 1.04–1.49 for ImC) and a tendency towards significance was observed for platelets (age-adjusted OR 1.08, 95% CI 0.98–1.19 for ImB and 1.03, 95% CI 0.93–1.14 for ImC) (Table 1). As a support of these findings, PLR counts were significantly negatively correlated with TIS (Figure 4C), in accordance with the notion that TIS metric portrays the presence of inflammatory molecules [46,47]. When comparing PLR and CYT, a trend towards a negative correlation was observed (Figure 4D). As expected, systemic lymphocytes were positively correlated with TIS scores (Figure 4E), since during inflammation the cellular microenvironment becomes highly reactive mainly due to T lymphocyte action [48].

No differences or statistical associations between the Im-Clus and the other hematological inflammatory markers (NLR, lymphocytes or neutrophils) were found (Table 1, Figure S3A–F). The different counts of PLR and platelets in patients with tumors belonging to different Im-Clus indicates that specific systemic hematological markers of inflammation correlate with local immune infiltrate at tumor site in TNBC cases. 

### 2.4. Prognostic Relevance of Local and Systemic Inflammatory Markers

**Figure 5:**
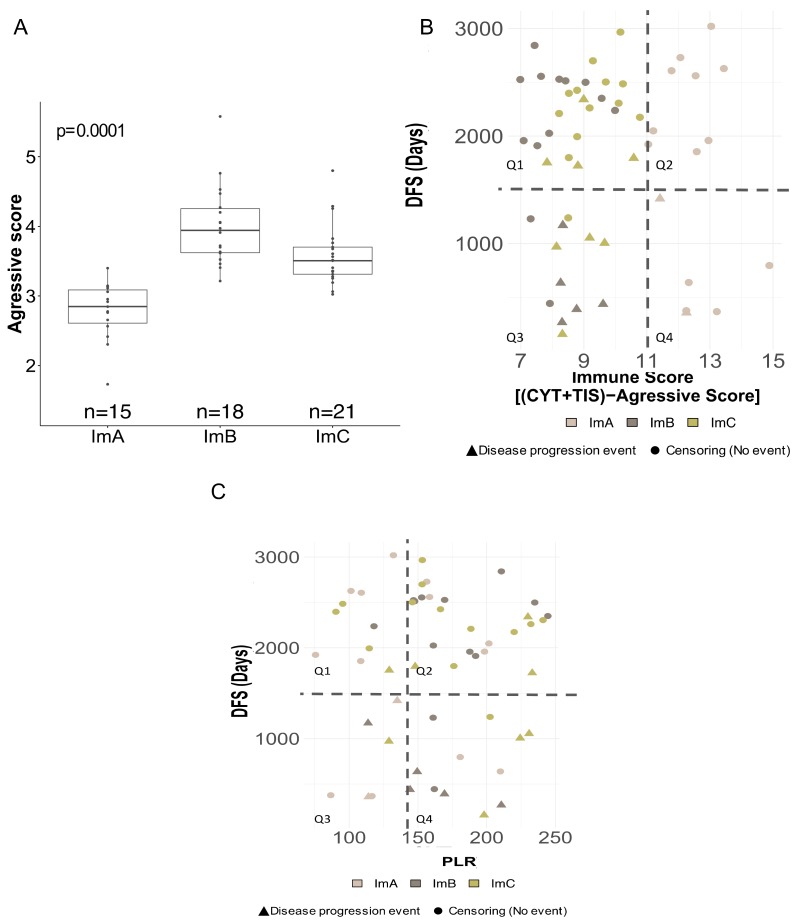
Clinical relevance of local and systemic inflammatory markers (**A**) Boxplot of computed aggressive score among Im-Clus (**B**) Scatterplot of follow-up time (y-axis, time to event (days), cut-off mean value) vs Integrative immune score (IMS score), IMS = ((CYT × TIS) − Aggressive score) (x-axis, cut off mean value). In each plot four different quadrants are indicated (Q1, Q2, Q3, Q4) in accordance to their value to y and x values. Circle represents no progression/recurrence event while triangle represents event. Colors represent each Im-Clus as indicated by the legend color. (**C**) Scatterplot of follow-up time (y-axis, time to event (days), cut-off mean value) vs. PLR values (x-axis, (cut-off mean value of ImA cluster).

None of the Im-Clus showed a significantly longer DFS outcome, probably due to the limited numbers of cases and progression events in each immuno-cluster that limit the statistical significance than ImB and ImC clusters (Figure S4). However, considering the recurrence events in each immune-clusters, we observed more events in patients whose tumors belong to ImB and ImC compared to ImA (Figure S4). To explore the clinical relevance of the Im-Clus we tested the aggressive score gene-expression signature [49], whose independent prognostic relevance has been proved previously [37,50,51]. In keeping with our previous immune-state characterization, the aggressive score also differed among the three immuno-clusters, with ImA tumors harboring the significantly lowest aggressive score, while ImB and ImC showed similar aggressive values (median: 2.85 vs. 3.94 and 3.51, *p* = 0.0001) (Figure 5A). Moreover, by analyzing an integrative immune score (IMS), that considers the biological cooperative activity of CYT and TIS phenotypes and the aggressive score measurement as a molecular surrogate indicator of TNBC outcome (see section of Materials and Methods for details), against the time to event (disease recurrence or censoring), all ImA tumors showed the highest IMS score and the highest event-free time (Figure 5B, Q2), notably only two ImA tumors harboring high IMS levels also presented cancer-disease event (Figure 5B, Q4). Tumors identified by the lowest IMS score and the earlier progression events were classified as ImB and C (Figure 5B, Q3). Even more, ImC tumors presenting late recurrent events were also characterized by a decreased IMS (Figure 5B, Q1).

The relationship between blood systemic inflammatory markers and clinical outcome was investigated. The association of PLR value, a marker of poor outcome in breast cancer, with aggressive score resulted near the significance (age-adjusted OR 2.08, 95% CI 0.69–6.30) (Table 2). Consistently, platelets were significantly associated with high aggressive score signature (age-adjusted OR 3.78, 95% CI 1.20–11.91) (Table 2). This analysis revealed that TNBCs with highest aggressive score and more abundant levels of platelets and PLR, are more likely to present poorer clinical outcomes. Further, the prognostic relationship between tumors with high CD8+ T cells score based on gene expression enrichment of the local tumor microenvironment (above median) showed less PLR levels in blood (age-adjusted OR 0.35 95%, CI 0.11–1.06). No association was detected between the tumors with high percentage of local TILs and blood platelets or lymphocytes (Table 3).

Comparing the PLR values with time to event, we observed that most of the patients with an early disease progression event, belonging to ImB and C groups, also present PLR levels above the ImA median value (Figure 5C, Q4), whereas a broad spectrum of progression times was observed in the rest of the quadrants (Figure 5C, Q1,2,3).

## 3. Discussion

Besides the already well-described molecular heterogeneity of TNBC tumor cells, the immune infiltrating counterpart has also emerged as a relevant contributor to define the tumor complexity [52,53]. A comprehensive portrait of immunologic landscape of breast cancer subtypes pointed out TNBC as the subtype with the strongest tumor immunogenicity [54]. Here, we have analyzed the immune states and the heterogeneity of infiltrating subpopulations in a well characterized TNBC series, integrating transcriptional-based deconvolution algorithms and effector-regulatory immune signatures. We find three robust immuno-clusters, with broad diversity in the tumor immune infiltrate composition and activation. Our data report that both cytolytic activity signature, reflecting an effective natural anti-tumor immunity, and tumor inflammation signature, denoting a T cell-inflamed microenvironment, are positively correlated with TILs among the immune-clusters. In agreement, in our TNBC series we observed a strong significant correlation between the percentage of TILs and the enrichment score of CD8+ T cells activation, the main driver of the primary antitumor immune-responses [55].

Neoplastic cells can interact with the immune cells infiltrating the tumor and activate adaptive processes, through modulation of transcriptional and signaling programs that may result in the establishment of an aggressive phenotype [51,56,57]. In keeping with immune-state characterization, the aggressive score also differed among the three immuno-clusters, with ImA tumors harboring a significantly lowest aggressive score, while ImB and ImC showed similar aggressive values. The differential capability of the immune system to mount durable antitumor immune responses, protective for metastasis and recurrence, is a clear evidence of the existence of immune subclasses in breast tumors [11,52,53]; on the other hand, tumors are able to mold the surrounding microenvironment to favor their own progression [58]. Several studies have shown a significant relationship between the number of TILs and recurrence-free survival in TNBC [15,43,59]. It has also been reported that complete pathological response following neoadjuvant chemotherapy is associated with higher TILs proportion in breast cancer patients [15,59]. Thus, TILs are today considered a prognostic factor and a predictive marker of chemotherapy response in TNBC. In the immunotherapy era, immune infiltrate characterization represents a hot topic for cancer research, especially for aggressive subtype such as TNBC, for which therapeutic options are limited. Collectively, our analysis indicates that decoding the quantitative and qualitative immune heterogeneity of the neoplastic tissues provides relevant information on the overall immune activation state at tumor site.

Recent studies have highlighted the complexity of the number and functional status of different immune networks [60,61], not only at local tumor level, but also at the systemic level, in the circulating blood tissue. Indeed, cancer-related systemic inflammatory responses are associated with alterations in circulating blood cells, mainly in their counts. Moreover, we recently demonstrated that BCs with aggressiveness features, such as TNBC, modify the surrounding microenvironment which contribute to the release of pro-inflammatory mediators [62].

Here, we found a robust association between immune clusters, representing subtypes of TNBC with different local immune features, and PLR values. Conversely, we did not find a significant association between any other hematological systemic cell count or cells ratio. Moreover, when dividing our cases based on the percentage of TILs infiltration, it is unlikely that tumors with higher percentage of TILs also present high PLR values. 

Low systemic lymphocyte counts can be associated with impaired activation of adaptive immunity in different tumors [63,64,65]. Conversely, high platelets indicate systemic inflammation and can be associated with increased metastatic processes of neoplastic cells [66,67,68,69]. The differentiation of megakaryocytes to platelets could be triggered by the tumor-associated inflammatory mediators, such as IL-1, IL-3, and IL-6 [70] that accelerate tumor cell growth and dissemination; in addition, platelets cooperate to protect circulating cancer cells from the immune system [71]. We also described the role of platelet-derived growth factor receptors beta (PDGFRβ) in mediating the endothelial differentiation of triple negative breast carcinoma cells [72] and we recently described the involvement of PDGFRβ in the regulation of the CDCP1 [73], a transmembrane protein which is overexpressed in TNBCs and is involved in tumor progression [74]. Hence, high PLR values may indicate an impaired host antitumor immune status. 

Growing evidence shows that PLR has clinical implications for the selection of therapeutic modalities and prognosis prediction for breast cancer patients [75,76,77] and PLR was recently associated with significantly lower progression-free survival in metastatic TNBC patients treated with carboplatin-paclitaxel or carboplatin-gemcitabine combination [78]. In agreement, our data suggest a slightly prognostic relationship between PLR value and highest aggressive score signature. As a support, negative correlations were observed between immune-signatures, percentage of TILs and aggressive score, suggesting that a less aggressive phenotype in TNBC is related to both local and systemic inflammatory features, closely connected to each other.

**Figure 6:**
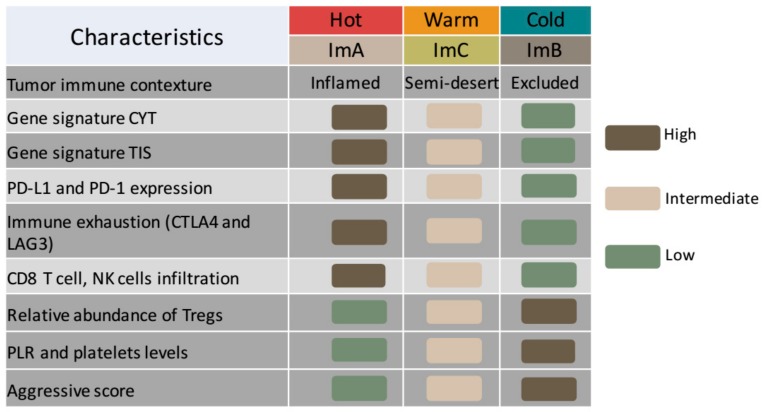
Each Immuno-cluster can be described as unique entity similar to cold, warm and hot immune tumors. Summary plot of the local and systemic immune characterization among the defined Im-Clus in our triple negative breast cancer (TNBC) cases.

Collectively, these data provide strong evidence of the existence of three immune-tumoral categories correlated with systemic hematological PLR levels in TNBC. Particularly, we clearly described an immune-active subtype (ImA) that is inversely correlated with PLR and platelet values and it is associated with favorable prognosis. ImA was characterized by “T cell–inflamed” phenotype as shown by type I IFN activation represented by TIS metric, cytotoxic effector molecules as shown by the CYT score and high CD8+/CD4+ T and NK cells infiltration. In addition, this cluster presented an enriched expression of immune inhibitory pathways (PD-L1/PD-1/CTLA4 axis) induced by inflammatory process. Conversely, it was found less enriched in Treg cells. This T cell–inflamed subtype presented the lowest aggressive score and a diminished number of progression events compared to the other Im-Clus. Considering all described features, these tumors can be described as “hot” tumors with a local immune landscape that may respond to immunotherapeutic and chemotherapeutic approaches (Figure 6) [79,80,81,82]. ImB tumors, by contrast, are similar to whose which are called “T cell excluded malignancies” or “cold tumors”, which do not show strong immune response. The result is an immunosuppressed phenotype with higher PLR systemic levels and increased progression events [79,80,81,82,83]. Finally, ImC tumors can be described as “warm” or excluded tumors since they present an intermediate phenotype with positive correlations between CYT and TIS scores, and a moderate expression of immune inhibitory pathways. ImC tumors present an intrinsic immune activity able to effectively mount a T cell-mediated response, but the tumor can escape such response [83] and present an intermediate PLR metric. 

Our findings agree with a prior study that described immune-landscape of TNBCs and identified subtypes based on immunogenic profiling through a ssGSEA and hierarchical clustering in different TNBC datasets [84]. The identified distinct subtypes (immunity high, medium and low) are comparable to our non-supervised approach that defined similar immune-clusters. These data further strengthen the concept and relevance of immune heterogeneity in TNBC. However, this study only analyzed local immune-environment and did not assess the correlation of hematological inflammatory markers.

There are certain limitations in the present study, including the relatively small sample size evaluated (*n* = 54) that may have influenced the partial clinical association observed between Im-Clus and time to disease progression. Moreover, this is a retrospective study conducted at a single institution that may not reflect other patient populations. Nonetheless, the presented strategy should serve as a proof-of-concept to show a potential step toward an improvement in real-time monitoring tools through routine blood tests that can provide information about local immunogenomic characteristics and the dynamic changes of tumor immune state during treatment, with a relevant impact on clinical decisions. This latter association could, after a robust validation in a larger cohort, improve the development of an effective and inexpensive approach that may help to guide the evaluation of local immune contexture by systemic immune markers.

## 4. Materials and Methods

### 4.1. Study Setting

A monocentric, retrospective study on patients with TNBC that receive surgery between 2002 and 2006 at Fondazione IRCCS Istituto Nazionale dei Tumori, Milan, Italy (baseline characteristics of the cases in Table S1) was performed. Histologic subtype and grade were determined according to WHO classification and Nottingham histologic grading system, respectively. An informed consent was obtained from all patients. All procedures were carried out in accordance with the Helsinki Declaration (World Medical Association, 2013) and the study was conducted after approval from the Institutional Review Board and the Independent Ethical Committee of Fondazione IRCCS Istituto Nazionale dei Tumori (Milan, Italy) (INT 160/15, 22-09-2015). Eligibility criteria were: (1) pathologically or cytologically confirmed diagnosis of primary non-metastatic TNBC, as defined by ER < 10% and PgR < 10% expression at immunohistochemistry (IHC) analysis and an IHC score for HER2 of 0 or 1+; (2) at least 60% tumor cell content evaluated by a pathologist; (3) availability of baseline (pre-surgery) absolute peripheral blood neutrophil, lymphocyte and platelet counts; (4) available information about previous treatment(s); (5) available information about previous treatment(s); (6) available information on the date of disease progression and patient death and (7) available information about gene expression profile. All subjects fulfilling these criteria were evaluated.

### 4.2. Evaluation of Systemic Inflammation Biomarkers 

For all evaluated tumors, CBC test data were available. Absolute counts of peripheral blood neutrophils, lymphocytes, platelets and monocytes were obtained from whole blood count samples taken within 2 months prior to primary tumor surgery for each patient by CBC test. Following parameters have been calculated: (a) NLR by dividing neutrophil by lymphocyte counts; (b) PLR by dividing platelet by lymphocyte counts. Blood parameters were evaluated before TNBC surgery.

### 4.3. Assessment of Intratumoral Inflammation

Hematoxylin and eosin-stained tissue sections of formalin fixed paraffin-embedded tumor specimens were collected for all patients. From this slide, a certified pathologist scored the average TILs density within tumor areas. Areas of adenoma, ulceration, and necrosis were excluded from the analysis. TILs density was calculated as the ratio of the area occupied by mononuclear cell infiltrates to the entire stromal area (% TIL = area occupied by mononuclear cells in tumor stromal/total stromal area) [85]. Pathologist was blinded from patient outcomes.

### 4.4. Transcriptional Landscape Analysis of TNBC

Global gene expression was assessed by the Human Transcriptome Array 2.0 platform (Affymetrix, Central Expressway, Santa Clara, CA, USA). The hybridization, washing and scan procedures were performed according to the protocol proposed by the manufacturer. RMA background correction and quantile normalization were performed using the Transcriptome Analysis Console Software (V2.0, Affymetrix, Santa Clara, CA, USA). Profiling data is available thorough Gene Expression Omnibus data repository (GEO) with accession number GSE86945.

### 4.5. Immuno-Clusters Identification 

We applied consensus clustering using non-negative matrix factorization (NMF) of selected immuno-related genes listed in the literature [17,19] on NMF bioconductor package [86] with Euclidean divergence on R environment (http://www.R-project.org). The initial number of mathematical clusters was selected based on the cophenetic correlation coefficient, which indicate the stability of the clusters. Then to get reliable robust biological clusters, we applied to strategies: (1) we first exclude the less represented cluster (C2; *n* = 3) since any relevant information from a mathematical point of view will be able to be obtained; (2) we then cluster together the most correlated groups, C4 and C5, as evaluated by Pearson correlation to get a more robust biological group, resulting in 3 distinctive clusters. 

### 4.6. TNBC Immune-Clusters Characterization by Immune Gene Signatures and CIBERSORT Analysis

The immunophenoscore score [19] was computed based on the gene expression values of immune-related genes to describe four classes of immune cells: (1) effector cells, (2) immunosuppressive cells, (3) MHC molecules and (4) selected immunomodulators. The immunophenoscore values were determinate with the available R-script deposited on GitHub (https://github.com/mui-icbi/Immunophenogram). ESTIMATE algorithm [21] was performed to infer the fraction of stromal and immune cells in the bulk gene expression profiles through the R code deposited on GitHub. The relative proportions of infiltrating immune cells were explores using the CIBERSORT [17] algorithm (http://CIBERSORT.stanford.edu/), using the default signature matrix and 1000 permutations. Cytolytic activity (CYT) was calculated through a validated gene expression signature based on the geometric mean of gene expression levels of granzyme A (GZMA) and perforin-1 (PRF1) [20]. Tumor Inflammation Signature (TIS) score was calculated as the average of continuous mean of log2-transformed normalized expression of the identified genes [18]. Calculation of Aggressive score was performed as described based on normalized expression levels of CCL5 (Chemokine (CC motif) ligand 5), DDIT4 (DNA-damage-inducible genes transcript 4) and POLR1C (Polymerase (RNA) I polypeptide C, 30 Kd1a) genes [49]: (− 0.393 × CCL5 + 0.443 × DDIT4 + 0.490 × POLR1C). Finally, individual enrichment scores for ssGSEA of CD8 activated T cell gene set [19] was quantified by ssGSEA method implemented in GSVA Bioconductor library [87] with min gene set size of 5 parameter. 

### 4.7. Statistical Analysis

The difference in clinical-pathological characteristics between Im-Clus was assessed by the Fisher’s exact test. Correlation between continuous variables was assessed by Spearman’s rank coefficient (ρ). Distributions of local and systemic inflammation markers within Im-Clus were illustrated by box plots and differences in median values were assessed by nonparametric Kruskal-Wallis rank test (* *p*-value ≤ 0.05; ** *p*-value ≤ 0.01; *** *p*-value ≤ 0.001; **** *p*-value ≤ 0.0001). Local and systemic inflammation markers were categorized according to their median value. Association between above median (high) systemic inflammation marker and Im_Clus was evaluated by multinomial logistic regression, with ImA as referent cluster. Associations between dichotomized aggressive score, local immune and systemic inflammation markers were evaluated by binomial logistic regression. Unadjusted (crude) and age-adjusted odds ratios (ORs), with 95% confidence intervals (CIs), were calculated. Time to event was defined as the time from surgery to the first event of locoregional recurrence, contralateral breast cancer, distant metastasis or the end of study. The Kaplan-Meier method was used to estimate 5-year disease free-survival (DFS) and differences between groups were assessed with the log-rank test. Being aware of the low number of our cases, to examine the disease progression in each Im_Clus according to tumor-immune signatures and systemic inflammation markers, we displayed scatter plots of the IMS integrative score (calculated as IMS = (CYT × TIS) − Aggressive score) and PLR/platelet count against time to disease progression or censoring. Two-sided *p* values < 0.05 were considered significant. Analyses were performed using the Stata statistical software, (release 12.0, Stata Corporation, College Station, TX, USA). Plots were generated in R environment with ggplot tool [88].

### 4.8. Data Availability

The datasets generated and/or analyzed during the current study are available from the corresponding author on request.

## 5. Conclusions

We identified three TNBC clusters displaying unique immune features. Deep molecular characterization revealed a TNBC cluster with a “T cell–inflamed” phenotype, a cluster defined as “T cell excluded malignancies” and a last cluster that presents an intermediate phenotype.

To the best of our knowledge, this is the first time that immune tumor characteristics have been related with systemic inflammatory parameters in BC. These data open the possibility to identify by a simple blood test certain subpopulations of immune-active TNBC tumors displaying an enriched expression of immune inhibitory pathways (PD-L1/PD-1/CTLA4 axis) induced by inflammatory process, that may contribute to differentiate responder patients to immune-checkpoint inhibitors therapy. As a support, both TIS and CYT gene signatures, associated with the identified Im-Clus, probe a good correlation with predictive effects of immunotherapy (anti PD-L1 response) in other tumors (melanoma, head and neck and gastric cancer) [18,20,89]. Overall, our immune characterization highlights the potential of hematic markers to mirror local tumor infiltrate in TNBC patients and could be exploited to decipher tumor infiltrate properties and consequently to select the most appropriate therapies.

## Figures and Tables

**Table 1:** Association between markers of systemic inflammation and immune-clusters.

Inflammation Markers	ImA	ImB	ImC
PLR			
median (range)	132.2 (75.6–210.0)	161.6 (113.8–244.5)	176.2 (90.4–241.0)
*Kruskal-Wallis* test	*p* = 0.045		
crude OR (95% CI) ^a,b^	Ref.	1.20 (1.00–1.43)	1.23 (1.04–1.47)
age-adjusted OR (95% CI) ^a,b^	Ref.	1.20 (1.00–1.44)	1.24 (1.04–1.49)
NLR			
median (range)	2.60 (1.05–4.46)	2.36 (1.14–4.11)	2.80 (1.08–9.67)
*Kruskal-Wallis* test	*p* = 0.963		
crude OR (95% CI) ^a,c^	Ref.	1.03 (0.60–1.77)	1.25 (0.76–2.05)
age-adjusted OR (95% CI) ^a,c^	Ref.	1.00 (0.54–1.86)	1.20 (0.67–2.16)
Lymphocytes			
median (range)	2.00 (0.90–3.90)	1.85 (0.90–2.60)	1.60 (0.70–3.40)
*Kruskal-Wallis* test	*p* = 0.265		
crude OR (95% CI) ^a,c^	Ref.	0.61 (0.21–1.78)	0.40 (0.13–1.25)
age-adjusted OR (95% CI) ^a,c^	Ref.	0.64 (0.22–1.87)	0.41 (0.13–1.31)
Neutrophils			
median (range)	4.40 (2.10–6.50)	4.55 (1.90–7.80)	4.10 (2.00–11.60)
*Kruskal-Wallis* test	*p* = 0.665		
crude OR (95% CI) ^a,c^	Ref.	0.99 (0.68–1.44)	0.95 (0.65–1.37)
age-adjusted OR (95% CI) ^a,c^	Ref.	0.93 (0.61–1.41)	0.87 (0.58–1.32)
Platelets			
median (range)	252 (119–452)	297 (169–489)	248 (132–521)
*Kruskal-Wallis* test	*p* = 0.060		
crude OR (95% CI) ^a,c^	Ref.	1.07 (0.97–1.19)	1.03 (0.93–1.13)
age-adjusted OR (95% CI) ^a,c^	Ref.	1.08 (0.98–1.19)	1.03 (0.93–1.14)

^a^ Odds ratios of ImB and ImC (ImA as reference) by increasing inflammation marker (continuous variable) estimated from multinomial logistic regression; ^b^ OR for each 10 unit increase in inflammation marker; ^c^ OR for each unit increase in inflammation marker. PLR: Platelet-to-Lymphocyte Ratio, CI: Confidence Interval, NLR: Neutrophil-to-lymphocyte ratio, OR: Odds ratio, Ref.: Reference.

**Table 2:** Association between markers of systemic inflammation and aggressive score.

Marker	*n*	High ^a^ Aggressive Score		
		(%)	Crude OR (95% CI) ^b^	Age-Adjusted OR (95% CI) ^b^
PLR				
≤median	27	40.7	Ref.	
>median	27	59.3	2.12 (0.71–6.27)	2.08 (0.69–6.30)
Lymphocyte				
≤median	28	50.0	Ref.	
>median	26	50.0	1.00 (0.34–2.91)	0.95 (0.31–2.83)
Platelet				
≤median	27	33.3	Ref.	
>median	27	66.7	4.00 (1.29–12.40)	3.78 (1.20–11.91)

^a^ Above median value; ^b^ Binomial logistic regression.

**Table 3:** Association between markers of systemic inflammation and tumor infiltrating lymphocytes (TILs).

Marker	High ^a^ Activated CD8 T Cell			High ^a^ TILs		
	(%)	Crude OR (95% CI) ^b^	Age-Adjusted OR (95% CI) ^b^	(%)	Crude OR (95% CI) ^b^	Age-Adjusted OR (95% CI) ^b^
PLR						
≤median	63.0	Ref.		40.7	Ref.	
>median	37.0	0.35 (0.11–1.04)	0.35 (0.11–1.06)	32.0	0.68 (0.22–2.14)	0.70 (0.22–2.24)
Lymphocyte						
≤median	42.9	Ref.		30.7	Ref.	
>median	57.7	1.81 (0.62–5.35)	1.89 (0.63–5.65)	42.3	1.65 (0.53–5.16)	1.79 (0.56–5.80)
Platelet						
≤median	63.0	Ref.		42.3	Ref.	
>median	37.0	0.35 (0.11–1.04)	0.36 (0.12–1.10)	30.8	0.61 (0.19–1.89)	0.65 (0.20–2.09)

^a^ Above median value; ^b^ Binomial logistic regression.

## Supplementary Materials

The following are available online at https://www.mdpi.com/2072-6694/11/7/911/s1, Table S1: Clinical-pathological characteristics of TNBC patients. Figure S1: Non-supervised clustering based on immune-related genes computed with non-negative matrix factorization (NMF) algorithm. Figure S2: Distribution of estimated tumor purities and proportion of samples infiltrated by specific immune cell population across immune-clusters. Figure S3: Distribution of systemic hematological inflammatory parameters that do not change (*p*-value > 0.05) among the Im-Clus (A) glucose level (B) L/M ratio (C) Neutrophils (D) Lymphocytes (E) Monocytes (F) N/L ratio, Figure S4: Kaplan Meir 5-year disease-free survival curve according to Im-Clus.
