# Supplementary material for: Decoding Immune Heterogeneity of Triple Negative Breast Cancer and Its Association with Systemic Inflammation
Source: Cancers (Basel). 2019 Jun 28;11(7):911. doi: 10.3390/cancers11070911 (PMC6678607; doi:10.3390/cancers11070911)
Supplement: Supplementary file 1 [file cancers-11-00911-s001.pdf]

Supplementary Materials: Decoding Immune Heterogeneity of Triple Negative Breast Cancer and Its Association with Systemic Inflammation

Sandra Romero-Cordoba, Elisabetta Meneghini, Milena Sant, Marilena V. Iorio, Lucia Sfondrini, Biagio Paolini, Roberto Agresti, Elda Tagliabue and Francesca Bianchi

Table S1. Clinical-pathological characteristics of TNBC patients.

Clinical Features	Total		ImA		ImB		ImC	
	n	% [a]	*n*	% [a]	*n*	% [a]	*n*	% [a]
Age, years								
mean (range)	53.2 (26–84)		47.7 (26–76)		54.3 (38–80)		56.2 (40–84)	
<50	20	37.0	8	53.3	8	44.4	4	19.1
≥50	34	63.0	7	46.7	10	55.6	17	80.9
					p [b] = 0.084			
Tumor size, mm								
<20	31	57.4	9	60.0	9	50.0	13	61.9
≥20	18	33.3	5	33.3	7	38.9	6	28.6
unknown	5	9.3	1	6.7	2	11.1	2	9.5
					p [b,c] = 0.759			
Lymph node metastasis								
no	31	57.4	10	66.7	11	61.1	10	47.6
yes	20	37.0	5	33.3	5	27.8	10	47.6
unknown	3	6.6	0	-	2	11.1	1	4.8
					p [b,c] = 0.484			
Grade								
well/moderately differentiated	9	16.7	1	6.7	2	11.1	6	28.6
poorly differentiated	44	81.5	14	93.3	16	88.9	14	66.7
unknown	1	1.8	0	-	0	-	1	4.8
					p [b,c] = 0.215			

[a] Column percentage; [b] Fisher's exact test; [c] Patients with no information excluded from test.

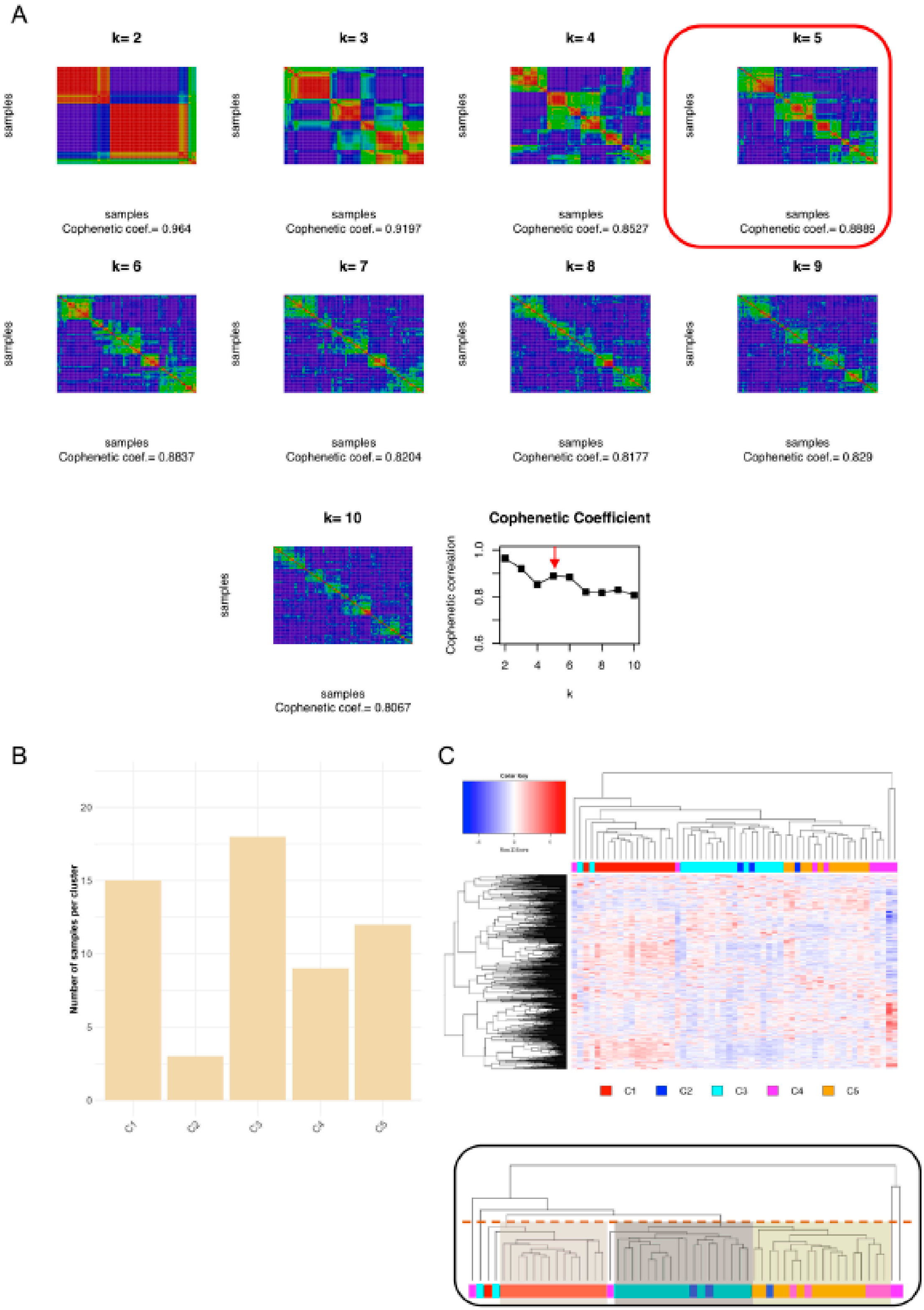

Figure S1. Non-supervised clustering based on immune-related genes computed with non-negative matrix factorization (NMF) algorithm. (**A**) Consensus NMF clustering identifies five robust subsets of immune tumor subgroups as shown by the cophenic distance plot (indicated by a red arrow) in our triple negative tumors (*n* = 54). (**B**) Bar plot showing the number of tumors belonging to each cluster. (**C**) Upper panel: Agglomerative semi-supervised hierarchical clustering of individual samples based on immune-related genes of the five subtypes resulted from the NMF clustering. Lower panel: dendogram depicting Pearson correlation among the five defined subtypes. Color squares showed the final clustering applied to our cases.

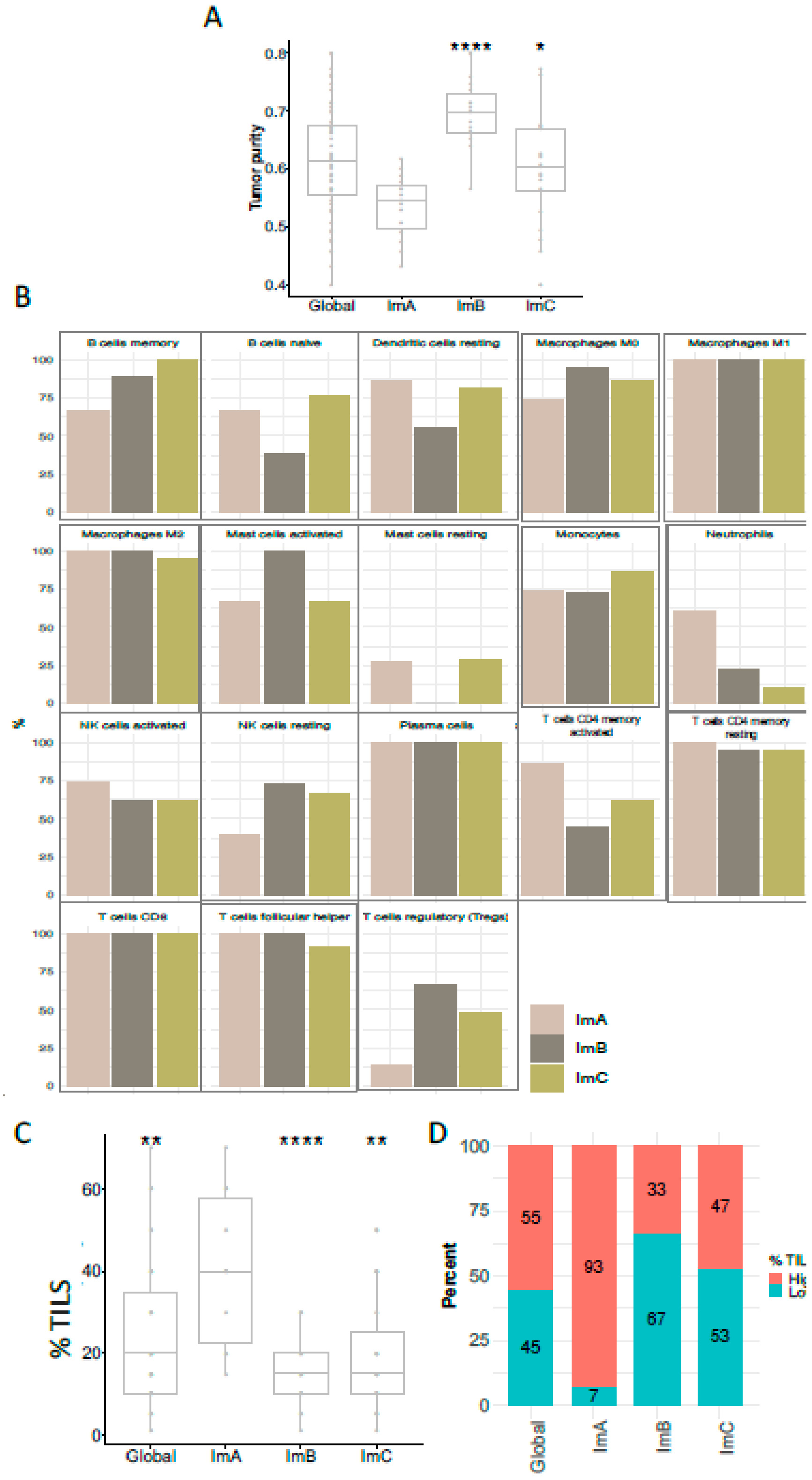

Figure S2. Distribution of estimated tumor purities and proportion of samples infiltrated by specific immune cell population across immune. (**A**) Boxplot comparing the tumor purity prediction of the entire cohort (Global) and of each Im-Clus. (**B**) Barplots representing the percentage of samples presenting at least 1% of the evaluated immune-populations computed by Cibersort. (**C**) Boxplot of the distribution of TILs percentage defined by the pathologist through eosin/hematoxylin evaluation. (**D**) Barplot showing the percentage of samples harboring high or low TILs % content (Cutoff ≥ 20%) in the TNBC cohort and Im-clus. Statistical comparison based on Kruskal-Wallis method. (* *p*-value ≤ 0.05; ** *p*-value ≤ 0.01; *** *p*-value ≤ 0.001; **** *p*-value ≤ 0.0001).

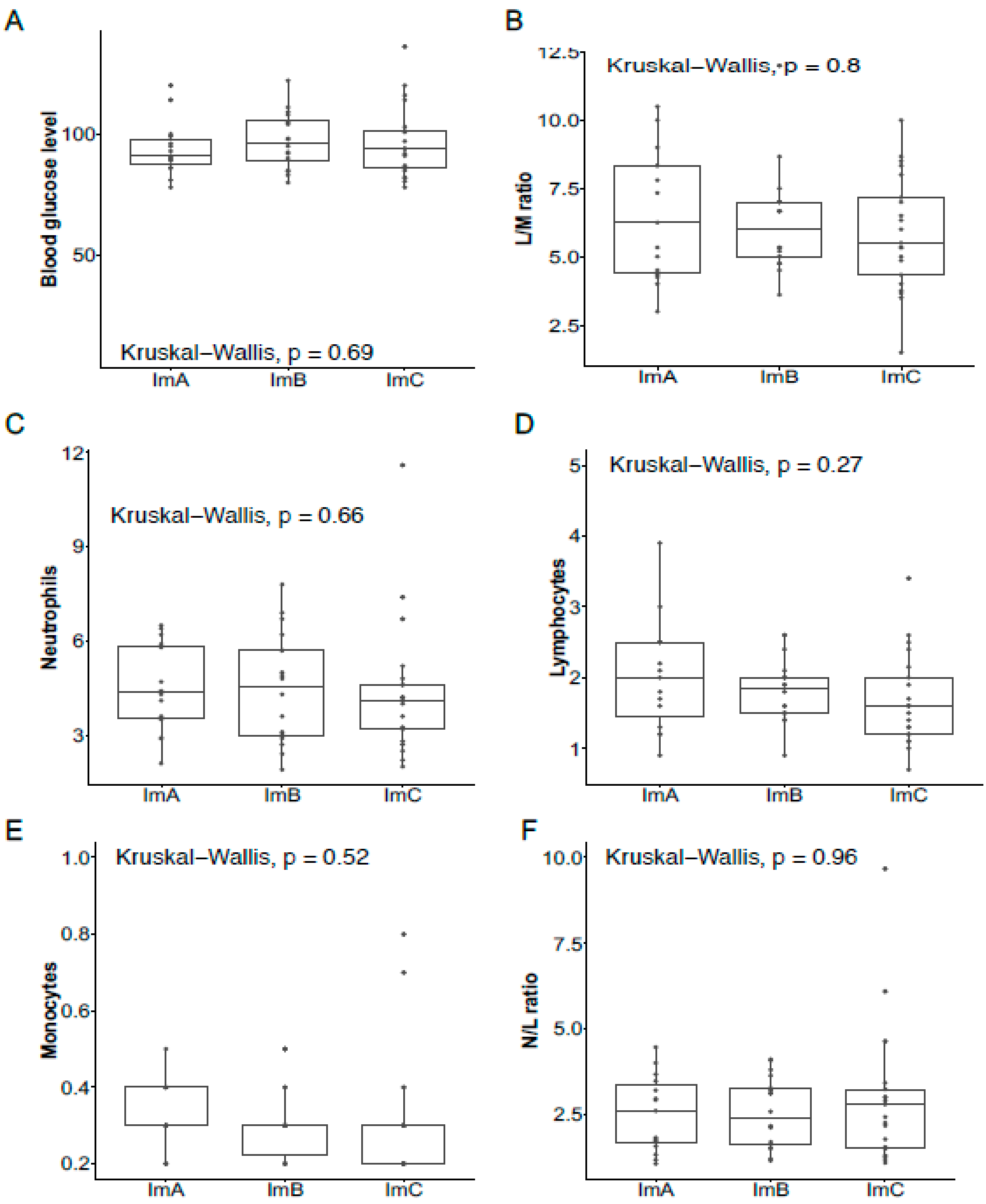

Figure S3. Distribution of systemic hematological inflammatory parameters that do not change (*p*-value > 0.05) among the Im-Clus (**A**) glucose level (**B**) L/M ratio (**C**) Neutrophils (**D**) Lymphocytes (**E**) Monocytes (**F**) N/L ratio. Statistical comparison based on Kruskal-Wallis method.

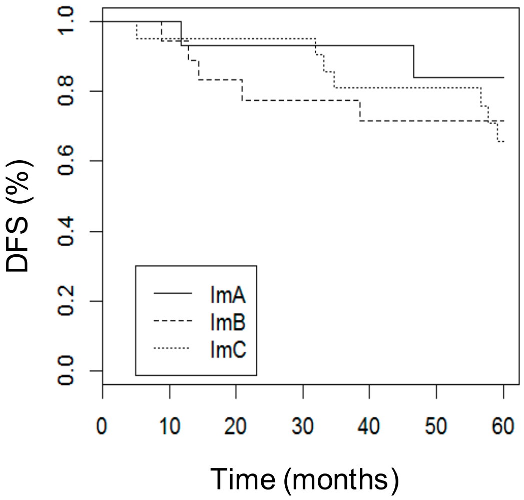

n. events	5-year DFS (95% CI)
2	84.0% (48.7-95.7%)
5	71.4% (44.3-87.0%)
7	65.8% (41.2-82.0%)
Log-rank	p=0.612

Figure S4. Kaplan-Meier 5-year disease-free survival curve according to Im-Clus.

© 2019 by the authors. Licensee MDPI, Basel, Switzerland. This article is an open access article distributed under the terms and conditions of the Creative Commons Attribution (CC BY) license (http://creativecommons.org/licenses/by/4.0/).
